# Effects of *N*-acetylcysteine on brain glutamate levels and resting perfusion in schizophrenia

**DOI:** 10.1007/s00213-018-4997-2

**Published:** 2018-08-23

**Authors:** Grant McQueen, John Lally, Tracy Collier, Fernando Zelaya, David J. Lythgoe, Gareth J. Barker, James M. Stone, Philip McGuire, James H. MacCabe, Alice Egerton

**Affiliations:** 1 Department of Psychosis Studies, Institute of Psychiatry, Psychology & Neuroscience, King’s College London, De Crespigny Park, London, UK; 2 Department of Psychiatry, Royal College of Surgeons in Ireland, Dublin, Ireland; 3 Department of Neuroimaging, Centre for Neuroimaging Sciences, Institute of Psychiatry, Psychology & Neuroimaging, King’s College London, De Crespigny Park, London, UK; 4 Experimental Medicine, Hammersmith Hospital, Imperial College London, London, UK

**Keywords:** Schizophrenia, Glutamate, *N*-Acetylcysteine, Magnetic resonance spectroscopy, Arterial spin labelling, Anterior cingulate cortex

## Abstract

**Rationale:**

*N*-Acetylcysteine (NAC) is currently under investigation as an adjunctive treatment for schizophrenia. The therapeutic potential of NAC may involve modulation of brain glutamate function, but its effects on brain glutamate levels in schizophrenia have not been evaluated.

**Objectives:**

The aim of this study was to examine whether a single dose of NAC can alter brain glutamate levels. A secondary aim was to characterise its effects on regional brain perfusion.

**Methods:**

In a double-blind placebo-controlled crossover study, 19 patients with a diagnosis of schizophrenia underwent two MRI scans, following oral administration of 2400 mg NAC or matching placebo. Proton magnetic resonance spectroscopy was used to investigate the effect of NAC on glutamate and Glx (glutamate plus glutamine) levels scaled to creatine (Cr) in the anterior cingulate cortex (ACC) and in the right caudate nucleus. Pulsed continuous arterial spin labelling was used to assess the effects of NAC on resting cerebral blood flow (rCBF) in the same regions.

**Results:**

Relative to the placebo condition, the NAC condition was associated with lower levels of Glx/Cr, in the ACC (*P* < 0.05), but not in the caudate nucleus. There were no significant differences in CBF in the NAC compared to placebo condition.

**Conclusions:**

These data provide preliminary evidence that NAC can modulate ACC glutamate in patients with schizophrenia. In contrast, physiological effects of NAC on the brain were not detectable as between session changes in rCBF. Future studies assessing the effects of a course of treatment with NAC on glutamate metabolites in schizophrenia are indicated.

## Introduction

Approximately two-thirds of patients with schizophrenia show a suboptimal symptomatic response to standard antipsychotic administration (Meltzer 1997; Lindenmayer 2000). There are substantial on-going efforts to develop alternative pharmacological interventions for this group, with a major focus on compounds that can modulate glutamatergic neurotransmission (Buchanan et al. 2007; Kinon and Gómez 2013; Bugarski-Kirola et al. 2016, 2017). The ability of such compounds to modulate glutamate levels can be tested in animal models during early stages of drug development (Moghaddam and Krystal 2012), but it is not known whether similar effects occur in patients with schizophrenia. This lack of studies evaluating target engagement in man is a major limiting factor in glutamatergic drug development for schizophrenia (Javitt et al. 2018).

*N*-Methyl-d-aspartate (NMDA) receptor antagonists, such as phencyclidine or ketamine, produce effects that resemble the symptoms of schizophrenia in man (Javitt and Zukin 1991; Krystal et al. 1994) and increase glutamate metabolite levels in frontal cortical brain areas (Moghaddam et al. 1997; Rowland 2005; Stone et al. 2012; Javitt et al. 2018). On this basis, glutamatergic theories of schizophrenia predict an elevation in cortical glutamate, and accordingly, a therapeutic potential for compounds that can facilitate NMDA function and decrease glutamate release (Moghaddam and Javitt 2012).

Brain glutamate concentrations can be measured in man using localised proton magnetic resonance spectroscopy (^1^H-MRS). This approach estimates concentrations of glutamate (Glu), its metabolite glutamine (Gln), and the combined measure of glutamate plus glutamine (Glx) in a pre-specified region of interest. ^1^H-MRS studies in people with schizophrenia or psychosis compared to healthy volunteers have reported both increases and decreases in regional glutamate levels, which may reflect illness stage, medication effects, or other methodological factors (Merritt et al. 2016). Recent studies indicate that glutamate levels in the anterior cingulate cortex (ACC) or striatum may be particularly elevated in patients who remain unwell after conventional antipsychotic treatment, compared to patients who have responded well (Egerton et al. 2012; Demjaha et al. 2014; Goldstein et al. 2015; Mouchlianitis et al. 2016). These findings may suggest that in patients who remain unwell despite antipsychotic treatment, elevated glutamatergic concentration in tissue is not reduced by conventional antipsychotic medication (Egerton et al. 2017) and that adjunctive interventions that can reduce glutamate levels may benefit this group. Evaluation of the ability of a glutamatergic compound to reduce ^1^H-MRS glutamate levels in schizophrenia may therefore provide a biomarker of target engagement, which could be used to facilitate clinical trials.

One compound which is currently under investigation for this purpose is N-acetylcysteine (NAC), which can decrease neuronal glutamate release (Anwyl 1999). In rats, PCP-stimulated increases in frontal glutamate are blocked by NAC pre-treatment (Baker et al. 2002), which could indicate its potential for use as an antipsychotic drug. NAC is a precursor to the amino acid l-cysteine (Arakawa and Ito 2007) and its oxidised form l-cystine, which is thought to modulate extracellular glutamate levels through interaction with the cystine/glutamate antiporter, System *x*_c_^−^ (Bridges et al. 2012a). System *x*_c_^−^ exchanges extracellular l-cystine with intracellular l-glutamate, most notably across the membrane of glial cells (Bridges et al. 2012b). Glutamate transported into the extracellular space through System *x*_c_^−^ activates extrasynaptic mGluR2/3 receptors (Baker et al. 2002; Xi et al. 2003; Mohan et al. 2011), to decrease neuronal glutamate release (Anwyl 1999). Accordingly, NAC’s ability to attenuate phencyclidine-stimulated glutamate increases appears to be mediated by a mGluR2/3-mediated mechanism (Baker et al. 2002). The effects of NAC on ^1^H-MRS measures of glutamate in people with schizophrenia have not been reported. However, in cocaine-dependent individuals, single-dose NAC administration decreases ACC ^1^H-MRS glutamate levels (Schmaal et al. 2012). This raises the possibility that NAC may also be able to decrease glutamate levels in patients with schizophrenia.

Another potential biomarker for antipsychotic efficacy may be drug-induced changes in regional brain activity, as measured through changes in resting regional cerebral blood flow (rCBF) due to the phenomenon of neuro-vascular coupling (Allen et al. 2016). Changes in rCBF have been observed after administration of single doses of antipsychotic compounds in both people with schizophrenia and in healthy volunteers (Lahti et al. 2003; Fernández-Seara et al. 2011; Handley et al. 2013; Shcherbinin et al. 2015). These studies have shown increases in CBF occurring in regions associated with the mechanism of action of the drug and relevant to schizophrenia, including the striatum, thalamus and ACC (Goozée et al. 2014). Arterial spin labelling (ASL) provides a modern MRI approach to measure rCBF and is well-suited to examining the changes in brain activity arising after administration of single oral doses of centrally acting compounds (Handley et al. 2013; Shcherbinin et al. 2015; Pollak et al. 2015). Changes in rCBF following NAC administration in regions previously associated with schizophrenia pathophysiology (Goozée et al. 2014) or with antipsychotic response (Tost et al. 2010; Handley et al. 2013) could therefore also indicate therapeutic potential.

The primary aim of the current study was to determine whether a single dose of NAC compared to placebo decreases brain glutamate metabolites in schizophrenia. Using ^1^H-MRS, we evaluated glutamate levels in the ACC and right caudate nucleus, the brain areas where an elevation in glutamate has been linked to a poor antipsychotic response (Egerton et al. 2012; Demjaha et al. 2014; Goldstein et al. 2015; Mouchlianitis et al. 2016), and where, in preclinical studies, the administration of NAC reduces glutamate levels (Baker et al. 2008; Durieux et al. 2015). The secondary aim was to use ASL to characterise the effects of NAC on rCBF in these regions, and to investigate whether these alterations are correlated with NAC-induced changes in glutamate metabolites.

## Methods

### Participants and clinical measures

This study received ethical approval from the NRES London-Harrow NHS ethics committee and was registered on clinicaltrials.gov, study number NCT02483130.

Twenty participants meeting DSM-IV criteria for schizophrenia were recruited from outpatient services within the South London and the Maudsley NHS Foundation Trust. Data from previous studies (Egerton et al. 2012; Demjaha et al. 2014) indicated that a sample size of 18 would be required to detect a 15% within-subject difference in ACC glutamate at 80% power and an alpha = 0.05, while a sample of 12 in each group provides 80% power to detect a 5% within-group difference in rCBF (Murphy et al. 2011; Handley et al. 2013).

Inclusion required written informed consent and good physical health, as determined by a physical health screen. Exclusion criteria included contraindications to MRI at 3 tesla or contraindications to NAC administration, including pregnancy, history of asthma, seizure, and drug or alcohol dependency. The study excluded patients with current or previous NAC or clozapine use. Symptom severity was assessed during the health screen using the Positive and Negative Syndrome Scale (PANSS; (Kay and Qpjer 1982)), Clinical Global Impression-Severity (CGI-S; (Guy, 1976)), and functioning was assessed using Global Assessment of Functioning scale (GAF; (Hall 1995)).

### Administration of *N*-acetylcysteine and placebo

The dose of NAC, and the time period between NAC administration and MRI scanning followed the previous study of Schmaal et al. (2012), which detected decreases in glutamatergic measures 1 h following 2400 mg NAC administration in cocaine-dependent individuals, as peak plasma levels of NAC occur 1–2 h after oral administration (Holdiness 1991). This single dose is also similar to that used in repeated administration in clinical trials of NAC in schizophrenia (Berk et al. 2008; Farokhnia et al. 2013; Conus et al. 2018). The order of NAC and placebo administration was block randomised such that an equal number of participants received either NAC or placebo in the first visit. MRI sessions were performed exactly 7 days apart and at the same time of day. The local pharmacy distributed blinded medication packs with visually identical appearing capsules for each session. Both the research team and the participants were blind to the order and nature of each administration.

### Magnetic resonance imaging

**Fig. 1:**
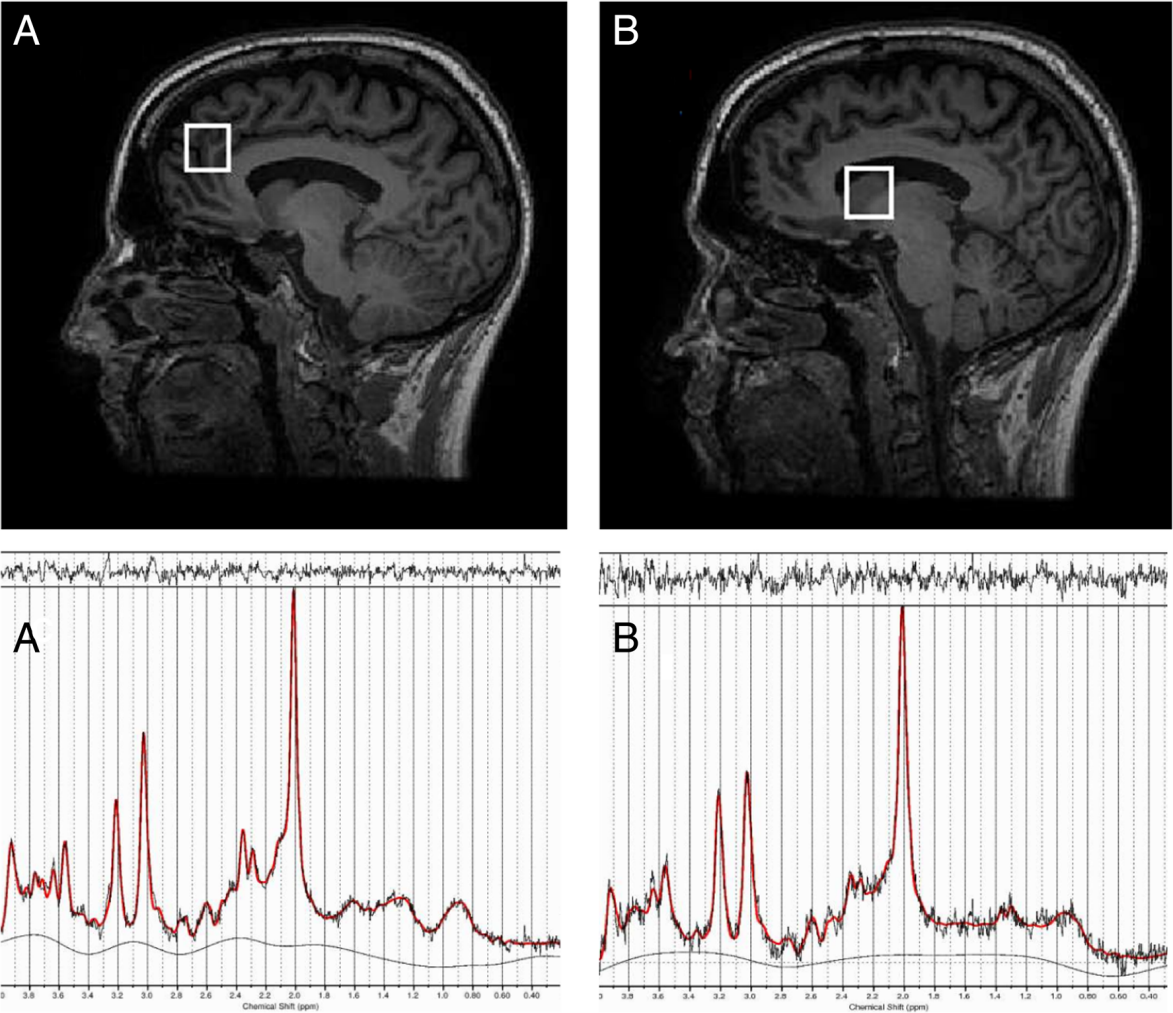
^1^H-MRS voxel positioning and example spectra in the anterior cingulate cortex (**a**) and right caudate nucleus (**b**)

MRI data were acquired on a 3-tesla MR750 scanner (General Electric, Chicago, USA). The scanning session commenced with a localizer, standard Axial T2-weighted fast spin echo scan (TR / TE = 4380 / 55.72) and a T1-weighted structural scan (TR / TE = 7.312 / 3.01). The T1-weighted acquisition was used to plan ^1^H-MRS voxel placement, and for calculation of ^1^H-MRS voxel tissue content. ^1^H-MRS spectra were acquired in 8 cm^3^ (2 × 2 × 2 cm^3^) voxels prescribed in the bilateral ACC and right caudate nucleus (Fig. 1). Spectra were acquired using a conventional PRESS (Point Resolved Spectroscopy) acquisition with 96 averages, TR = 3000 ms and with a TE = 30 ms in the ACC (Egerton et al. 2012) and a TE = 35 ms in the caudate (De la Fuente-Sandoval et al. 2011). An additional 16 averages were acquired without water suppression for subsequent eddy current correction and water scaling. The acquisition used the standard GE PROBE (Proton Brain Examination) sequence with CHESS (Chemically Selective Suppression) water suppression. For measurement of regional CBF, a 3D pseudo-continuous ASL (pCASL) acquisition was used. Arterial blood was labelled using a long (1.5 s) train of adiabatic radio frequency pulses. After a post-labelling delay of 1.5 s, perfusion images were acquired with a 3D Fast Spin Echo (FSE) stack-of-spirals multi-shot readout (TE / TR = 32 ms / 5500 ms; ETL = 64) (Dai et al. 2008; Handley et al. 2013). CBF maps were computed in physiological units of millilitre blood per 100 mg of tissue per minute, with a voxel size of 1 × 1 × 3 mm^3^.

### Image processing

^1^H-MRS spectra were analysed with LCModel version 6.3-0I (Provencher 1993) using the standard basis set of 16 metabolites (l-alanine, aspartate, creatine, phosphocreatine, GABA, glucose, glutamine, glutamate, glycerophosphocholine, glycine, myo-inositol, l-lactate, *N*-acetylaspartate, *N*-acetylaspartylglutamate, phosphocholine, taurine) provided with LCModel, for the same field strength, PRESS sequence and echo times. Spectral quality was determined by visual inspection, as well as through values for spectral linewidth, signal-to-noise ratio and the Cramér Rao Lower Bounds of the individual metabolite estimates. To calculate ^1^H-MRS voxel tissue content, the T1-weighted structural images were segmented into grey matter, white matter and cerebrospinal fluid (CSF) using Statistical Parametric Mapping 8 (SPM-8) (http//www.fil.ion.ucl.ac.uk/spm) running in MATLAB 6.5 (MathWorks Inc. Sherbon MA, USA). GE Sage 7 (GE Medical Systems) was used to locate the coordinates of each voxel, which was then mapped on to the segmented structural image using in-house software. For consistency with the study of Schmaal et al. (2012), glutamate and Glx signals referenced to creatine were used for the primary statistical analyses, and we also report water-scaled metabolite levels corrected for voxel tissue content. Metabolite values were corrected for voxel tissue content using the formula Mcorr = *M* × (wm + 1.21 × gm + 1.55 × CSF) / (wm + gm), where *M* is the uncorrected metabolite, and wm, gm and CSF indicate the fraction of white and grey matter and cerebrospinal fluid content in the voxel. The formula assumes a CSF water concentration of 55,556 mol/m^3^ with the LCModel default brain water concentration of 35,880 mol/m^3^ (Kreis et al. 1993; Gasparovic et al. 2006).

Computation of the CBF values was done in the scanner following the methodology outlined in the recent ASL consensus paper (Alsop et al. 2015). Individual CBF maps were then transformed to the reference space of the Montreal Neurological Institute (MNI) using the Automatic Software for ASL Processing (ASAP) toolbox (Mato Abad et al. 2016) running in SPM-8 under Matlab 6.5. Default pre-processing options were used for skull-stripping, co-registration to the subject’s 3D anatomical scan, and normalisation to the MNI template based on unified segmentation. The normalised maps were finally smoothed using an 8-mm kernel.

### Statistical analysis

The effects of NAC on ^1^H-MRS metabolite levels were determined using paired samples *t* tests in SPSS software (version 22.0, Chicago, Illinois). For Glu/Cr and Glx/Cr in each voxel, the threshold for statistical significance was defined as *p* < 0.05, and the effect size was calculated as Cohen’s *d* for significant values. The effects of NAC on rCBF were determined using within-subjects, second-level analysis implemented in SPM-8. For rCBF region of interest (RoI) analysis, ACC and caudate masks were created using the Talairach Daemon database atlas implemented in SPM (Lancaster et al. 1997, 2000). Spearman’s correlation coefficients were used to explore relationships between the absolute change (NAC minus placebo) in Glu/Cr, Glx/Cr and the absolute change in rCBF in the ACC or caudate nucleus.

Finally, to explore potential effects of NAC on regional CBF, we conducted a whole brain voxel-wise search at *P* < 0.001 uncorrected for multiple comparisons, and then used cluster-based FDR correction for statistically significant clusters accepted at *P* < 0.05.

## Results

### Sample characteristics

Participant demographic and clinical measures are presented in Table 1. MRI datasets were available in 19 of the 20 included participants, as one patient experienced nausea and vomiting 1 h after NAC administration and did not complete the scanning session. The remaining 19 participants reported no adverse effects after either NAC or placebo administration. Of the 19 included participants, seven participants were currently receiving olanzapine, four risperidone, two aripiprazole, two paliperidone, one haloperidol, one zuclopenthixol, and one flupentixol, and one was currently not taking any antipsychotic medication. Participants were receiving antipsychotic medication from 4 months to 19 years (mean (SD) months = 83.69 (71.30)) and had an illness duration of 4 months to 30 years (mean (SD) months = 152.00 (93.56)). Eight participants were additionally receiving citalopram (2), procyclidine (2), olanzapine (1), venlafaxine (1), propanol (1), and sodium valproate (1).

**Table 1:** Subject demographics and clinical measures. Data are reported as mean (standard deviation). *CGI* Clinical Global Impression score, *GAF* Global Assessment of Functioning score, *PANSS* Positive and Negative Syndrome Scale scores

Age, years	41.57 (10.87)
Gender, female/male	3/17
Education, years	13.89 (1.85)
PANSS–positive	12.72 (4.17)
PANSS–negative	13.72 (3.85)
PANSS–general	23.11 (6.06)
PANSS–total	49.56 (12.27)
CGI	3.00 (0.57)
GAF	59.31 (4.79)

### ^1^H-MRS spectral quality

Due to a scanner fault, one ACC dataset was not collected, leaving 18 ^1^H-MRS datasets in the ACC and 19 ^1^H-MRS datasets in the caudate nucleus available for analysis. For all reported metabolites, individual Cramér Rao Lower Bounds were below 20% and no spectra were excluded on the basis of poor quality. There were no significant differences in values relating to ^1^H-MRS data quality or voxel tissue content in the placebo compared to NAC condition (Table 2).

**Table 2:** Estimates of linewidths (ppm), signal-to-noise ratios, Cramér Rao Lower Bounds, and voxel white matter, grey matter and cerebrospinal fluid fractions in the anterior cingulate cortex and right caudate nucleus in placebo and NAC conditions. Data are presented as mean (standard deviation). *Cr* creatine, *CSF* cerebral spinal fluid, *Glu* glutamate, *GM* grey matter, *Glx* glutamate + glutamine, mI myo-inositol, *NAA N*-acetyl aspartate, *S/N* signal to noise, *TCho* total choline, *WM* white matter

Spectral quality measures				
	Anterior cingulate cortex			
	Placebo	NAC	Statistic	
Linewidth	0.04 (0.01)	0.04 (0.01)	*t*(17) = 1.51, *P* = 0.14	
S/N	22.7 (5.40)	22.05 (3.90)	*t*(17) = 0.61, *P* = 0.54	
WM	0.15 (0.10)	0.15 (0.09)	*t*(17) = 0.13, *P* = 0.90	
GM	0.56 (0.10)	0.57 (0.12)	*t*(17) = − 0.32, *P* = 0.75	
CSF	0.29 (0.12)	0.28 (0.08)	*t*(17) = 0.26, *P* = 0.80	
	Right caudate nucleus			
Linewidth	0.07 (0.01)	0.07 (0.01)	*t*(18) = − 0.56, *P* = 0.57	
S/N	22.72 (5.43)	22.05 (3.96)	*t*(18) = 1.93, *P* = 0.68	
WM	0.49 (0.09)	0.47 (0.06)	*t*(18) = 0.81, *P* = 0.43	
GM	0.48 (0.11)	0.51 (0.05)	*t*(18) = − 1.05, *P* = 0.31	
CSF	0.03 (0.03)	0.02 (0.02)	*t*(18) = 0.93, *P* = 0.37	
Cramér Rao Lower Bounds (%)				
	Anterior cingulate cortex			
Glu	6.66 (2.02)	6.77 (1.51)	*t*(17) = − 0.19, *P* = 0.85	
Glx	6.50 (1.09)	7.44 (2.12)	*t*(17) = − 1.88, *P* = 0.77	
NAA	3.22 (0.42)	3.00 (0.76)	*t*(17) = 1.07, *P* = 0.29	
TCho	3.16 (0.51)	3.07 (0.62)	*t*(17) = 0.55, *P* = 0.58	
MI	5.27 (1.87)	5.00 (1.02)	*t*(17) = 0.92, *P* = 0.36	
Cr	2.88 (0.58)	2.77 (0.54)	*t*(17) = 0.69, *P* = 0.49	
	Right caudate nucleus			
Glu	8.05 (1.22)	8.21 (2.34)	*t*(18) = − 0.30, *P* = 0.76	
Glx	10.26 (2.30)	10.26 (5.26)	*t*(18) = 0.00, *P* = 1.0	
NAA	3.36 (0.68)	3.21 (0.91)	*t*(18) = 5.90, *P* = 0.56	
TCho	3.47 (0.51)	3.47 (0.51)	*t*(18) = 1.00, *P* = 1.00	
MI	8.47 (1.98)	9.15 (3.28)	*t*(18) = − 1.58, *P* = 0.13	
Cr	3.00 (0.33)	3.00 (0.33)	*t*(18) = 0.43, *P* = 0.66	

### Effect of NAC on voxel glutamate levels

**Fig. 2:**
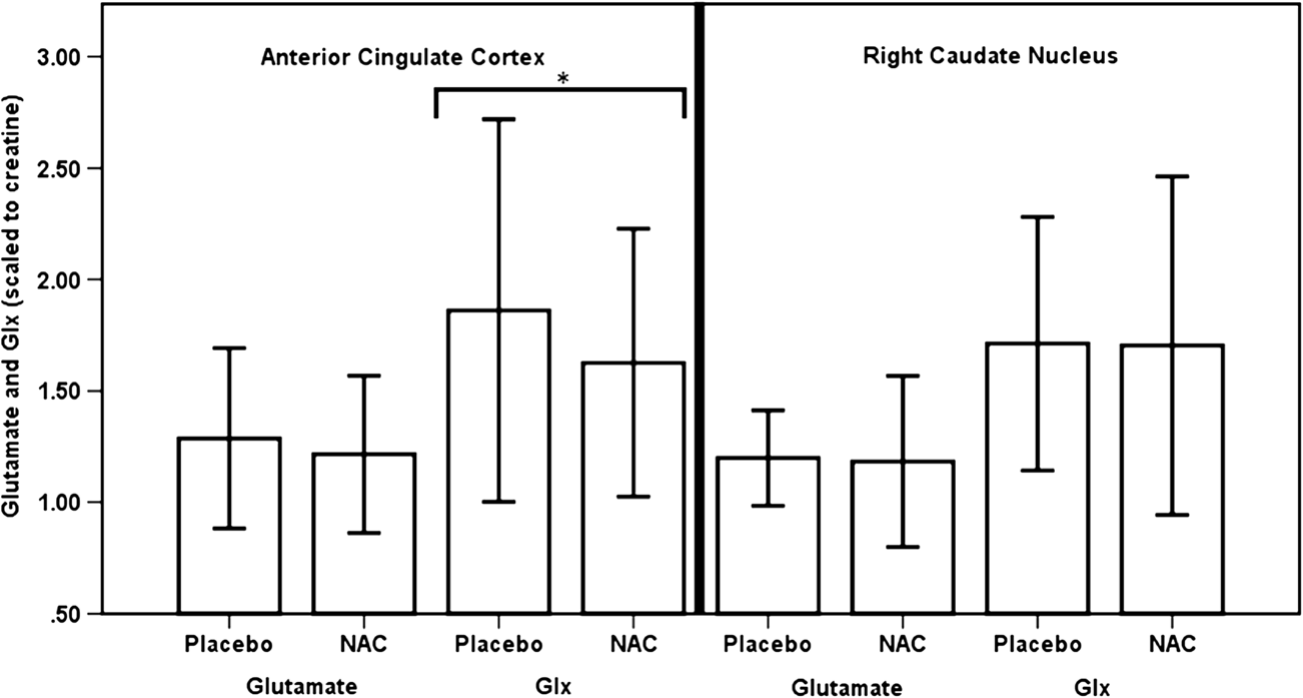
Glutamate and Glx (glutamate and glutamine) metabolite levels in the anterior cingulate cortex and right caudate nucleus, scaled to creatine. Data are presented as mean values, with standard deviation denoted by error bars. *Significantly lower levels of Glx/Cr in the ACC in the NAC compared to placebo condition (*p* < .05)

Compared to the placebo condition, the NAC condition was associated with significantly lower levels of Glx/Cr in the ACC (*t*(17) = 2.40; *P* = .03, *d* = 0.64; Fig. 2, Table 3). There were no significant effects of condition on Glu/Cr in the ACC, on Glx/Cr or Glu/Cr in the right caudate nucleus (Fig. 2), or on any of the other metabolites quantifiable from the ^1^H-MRS spectra in either region (Table 3).

**Table 3:** Creatine and water-referenced, CSF-corrected metabolite values in the anterior cingulate cortex and right caudate nucleus. Data are presented as mean, standard deviation and *t* test. *Cr* creatine, *Glu* glutamate, *Glx* glutamate + glutamine, *mI* myo-inositol, *NAA N*-acetyl aspartate, *TCho* total choline

Metabolite	Placebo	NAC	Statistic
Referenced to creatine			
Anterior cingulate cortex			
Glu/Cr	1.29 (0.20)	1.22 (0.18)	*t*(17) = 1.30, *P* = 0.21
Glx/Cr	1.86 (0.43)	1.63 (0.30)	*t*(17) = 2.40, *P* = 0.03*
NAA/Cr	1.31 (0.11)	1.28 (0.13)	*t*(17) = 0.86, *P* = 0.40
TCho/Cr	0.26 (0.03)	0.25 (0.02)	*t*(17) = 0.34, *P* = 0.74
MI/Cr	0.72 (0.10)	0.74 (0.14)	*t*(17) = − 0.81, *P* = 0.43
Right caudate nucleus			
Glu/Cr	1.20 (0.11)	1.19 (0.19)	*t*(18) = 0.20, *P* = 0.85
Glx/Cr	1.69 (0.29)	1.70 (0.37)	*t*(18) = − 0.05, *P* = 0.96
NAA/Cr	1.28 (0.16)	1.22 (0.11)	*t*(18) = 1.44, *P* = 0.17
TCho/Cr	0.26 (0.03)	0.26 (0.02)	*t*(18) = 1.31, *P* = 0.21
MI/Cr	0.51 (0.10)	0.49 (0.10)	*t*(18) = 0.89, *P* = 0.38
Corrected for voxel tissue content			
Anterior cingulate cortex			
Glu	14.67 (2.67)	14.77 (2.67)	*t*(17) = − 0.12, *P* = 0.90
Glx	22.55 (6.33)	19.80 (4.13)	*t*(17) = 1.92, *P* = 0.07
NAA	14.96 (2.16)	15.47 (1.28)	*t*(17) = − 0.87, *P* = 0.39
TCho	2.94 (0.52)	3.12 (0.49)	*t*(17) = − 1.30, *P* = 0.21
MI	8.19 (1.53)	8.97 (1.84)	*t*(17) = − 1.85, *P* = 0.82
Cr	11.48 (1.64)	12.19 (1.13)	*t*(17) = − 1.68, *P* = 0.11
Right caudate nucleus			
Glu	11.20 (1.47)	12.66 (6.04)	*t*(18) = − 0.98, *P* = 0.33
Glx	15.87 (3.24)	18.68 (12.00)	*t*(18) = − 0.98, *P* = 0.34
NAA	11.95 (1.75)	12.82 (5.26)	*t*(18) = − 0.67, *P* = 0.51
TCho	2.48 (0.39)	2.76 (1.31)	*t*(18) = − 0.88, *P* = 0.45
MI	4.73 (0.78)	5.13 (2.13)	*t*(18) = − 0.77, *P* = 0.45
Cr	9.43 (1.46)	10.62 (4.36)	*t*(18) = − 1.07, *P* = 0.30

### Regional cerebral blood flow

There was no significant difference in rCBF in the ACC (mean (SD) placebo = 47.22 (8.81); NAC = 46.83 (7.29); *t*(18) = .349, *P* = .73) or in the right caudate nucleus (mean (SD) placebo = 37.51 (7.48); NAC = 37.77 (6.71); *t*(18) = − .310, *P* = .76) in the NAC compared to placebo condition. Similarly, whole brain voxel-wise analysis found no significant differences in CBF between conditions. There was also no significant difference in global CBF between conditions (mean (SD) placebo = 39.64 (10.02); NAC = 40.03 (9.13); *t*(18) = − .398, *P* = .70).

### Relationship between glutamate metabolites, rCBF, and PANSS

There were no significant correlations between change in Glx/Cr, Glu/Cr and change in rCBF in either the ACC or caudate (all *P* > .6). There were also no significant correlations between PANSS total, positive and negative scores, and the change in Glu/Cr and Glx/Cr (all *P* > .1), or Glu/Cr and Glx/Cr in the placebo and NAC conditions in the ACC or caudate (all *P* > .3).

## Discussion

This study examined the effects of a single dose of *N*-acetylcysteine (NAC) on brain glutamate levels and resting perfusion in patients with schizophrenia. Glx/Cr in the anterior cingulate cortex (ACC) were significantly lower following administration of NAC compared to the placebo, but no further differences in the other glutamate metabolite measures in the ACC or in the caudate nucleus were apparent. The NAC compared to placebo condition was not associated with significant differences in cerebral blood flow (CBF).

The lower levels of ACC Glx/Cr after NAC compared with placebo administration in patients with schizophrenia is broadly consistent with a previous report of lower ACC glutamate following a single dose of NAC in cocaine-dependent individuals (Schmaal et al. 2012). That study described significantly lower levels of glutamate (in both creatine-scaled and water-referenced values), and a trend for lower levels of Glx after NAC administration in cocaine-dependent subjects but not in healthy controls, while we detected lower levels of Glx/Cr in the NAC condition in patients with schizophrenia. Although these results are similar, the different populations in these studies likely show different underlying neuropathology or neurobiological changes resulting from previous medication or drug use, which may have influenced sensitivity to NAC. It is also possible that the difference we see in Glx/Cr could be related to changes in creatine. However, CSF-scaled Glx levels showed a similar trend for lower levels in the NAC condition (*P* = .07). Our results thus provide preliminary evidence that NAC may decrease ACC glutamate metabolites in schizophrenia, but that will require further confirmation and replication. In addition, future studies will need to determine the extent to which lower levels of glutamate metabolites following a single dose of a glutamatergic compound are indicative of efficacy in improving symptoms over longer-term administration.

One methodological limitation of the ^1^H-MRS approach that we applied is that the glutamine signal cannot be reliably estimated. We were therefore unable to determine the relative contributions of glutamine and glutamate to the lower levels of Glx/Cr observed on NAC administration, although this would be possible in future studies using ^1^H-MRS optimised for detection of glutamate and glutamine. Furthermore, as our study did not include a baseline scan, we are unable to determine the relative effects of NAC and placebo administration on resting ^1^H-MRS metabolite levels or rCBF. A more general limitation of ^1^H-MRS is that as the signal reflects the total amount of glutamate in the voxel, it is not possible to ascribe the effects we observed to changes in glutamate neurotransmission specifically. Previous research indicates that NAC increases extracellular glutamate, which is then thought to decrease neuronal glutamate release via activation of presynaptic mGluR2/3 receptors (Baker et al. 2002). Thus, changes in the ^1^H-MRS signal may reflect the net effect of both increases in extra-synaptic and decreases in intra-synaptic glutamate following NAC administration. Indeed, in non-human primates, a decrease in binding of the mGluR5 radiotracer [^11^C] ABP688 following NAC may reflect increases in extra-synaptic glutamate levels (Miyake et al. 2011; Sandiego et al. 2013).

An additional limitation of our study is that the precise mechanism by which NAC may modulate the ^1^H-MRS glutamate signal is unclear. NAC can reduce neuronal glutamate release (Anwyl 1999) and modulate glutamate through System *x*_c_^−^ and mGluR2/3 signalling (Baker et al. 2002; Bridges et al. 2012b). However, NAC increases glutathione (GSH) (Berk et al. 2013), which decreases nitrosative stress (a marker of glutamate dysfunction) through redox modulation whilst reducing NMDA activity (Berk et al. 2013; Samuni et al. 2013). Therefore, GSH and NMDA modulation may contribute to the ^1^H-MRS glutamate signal after NAC administration.

A recent meta-analysis of ^1^H-MRS studies in schizophrenia suggests that there is no overall elevation in glutamate or Glx levels in the ACC/medial frontal cortex, but an increase in caudate glutamate and Glx levels, when compared to healthy controls (Merritt et al. 2016). Further studies have shown that ACC and caudate glutamate metabolites may be particularly elevated in patients in whom antipsychotic medication is ineffective (Egerton et al. 2012, 2017; Demjaha et al. 2014; Goldstein et al. 2015; Mouchlianitis et al. 2016).

These observations raise the possibility that the effects of NAC might be more marked in patients who have not responded to conventional treatment and have elevated glutamate levels. The current sample was not selected on the basis of antipsychotic response and had relatively low PANSS scores, which may therefore have limited the potential to observe a reduction in glutamate on NAC administration. Future studies could compare the effects of NAC or other glutamatergic interventions on glutamate levels in good versus poor antipsychotic responders.

Our second finding was that the NAC compared to placebo condition was not associated with significant differences in resting state perfusion in either the ACC or in the caudate, or in any other brain region. Previous studies that have used the same perfusion imaging approach have detected changes in regional perfusion after administration of single doses of several centrally acting compounds, including aripiprazole, haloperidol (Handley et al. 2013), risperidone (Shcherbinin et al. 2015), ketamine (Pollak et al. 2015), psilocybin (Carhart-Harris et al. 2012) and fentanyl (Zelaya et al. 2012). However, these studies involved comparison of data from drug and placebo sessions conducted on the same day, and studies where subjects were scanned on separate days have not found significant effects of lamotrigine or ketamine (Murphy et al. 2011; Shcherbinin et al. 2015). Assessment over two separate days may therefore have contributed to the absence of an observable effect of NAC on regional perfusion in our study. At similar doses and time periods after administration, NAC can reduce brain glutamate in cocaine-dependent patients (Schmaal et al. 2012) and reduce frequency-deviant mismatch negativity amplitude and increase P300 amplitude in healthy volunteers (Gunduz-Bruce et al. 2012). In addition, differences in brain network connectivity are observed after 4 days of administration of 2400 mg NAC during tobacco smoking abstinence (Froeliger et al. 2015). It thus seems unlikely that the lack of difference in perfusion following NAC reflects the sub-threshold doses or plasma levels. Nonetheless, the lack of any correlation between the change in Glx, glutamate, and rCBF indicates that the difference in ACC Glx/Cr following NAC administration is not driven by a difference in CBF. Alternative approaches, such as examining the effects of NAC versus placebo on rCBF on the same day, or the effects of NAC on resting state brain network functional connectivity may better reveal effects on brain neurophysiology.

Early clinical trials of adjunctive administration of NAC in schizophrenia suggest that it may have therapeutic effects (Lavoie et al. 2008; Berk et al. 2008, 2011; Bulut et al. 2009; Farokhnia et al. 2013; Dean et al. 2015; Conus et al. 2018). Our data provide preliminary evidence that these effects may be related to changes in brain glutamate levels. However, our findings are based on a single dose of NAC, and ^1^H-MRS imaging studies of longer-term NAC administration in schizophrenia incorporating efficacy measures are indicated. As discussed above, it would also be useful if future studies stratified patient samples by antipsychotic response, as the effects of NAC may be more evident in patients in whom conventional treatment is ineffective.
